# High SPIN4 Expression Is Linked to Advanced Nodal Status and Inferior Prognosis in Nasopharyngeal Carcinoma Patients

**DOI:** 10.3390/life11090912

**Published:** 2021-09-01

**Authors:** Shih-Lun Chang, Ti-Chun Chan, Tzu-Ju Chen, Ching-Chieh Yang, Hsin-Hwa Tsai, Cheng-Fa Yeh, Sung-Wei Lee, Hong-Yue Lai

**Affiliations:** 1 Department of Otolaryngology, Chi Mei Medical Center, Tainan 710, Taiwan; c3224710@ms16.hinet.net; 2 Department of Optometry, Chung Hwa University of Medical Technology, Tainan 717, Taiwan; 3 Department of Medical Research, Chi Mei Medical Center, Tainan 710, Taiwan; 090807@nhri.edu.tw (T.-C.C.); livelychord.tsai@biocheck.com.tw (H.-H.T.); 4 National Institute of Cancer Research, National Health Research Institute, Tainan 704, Taiwan; 5 Department of Clinical Pathology, Chi Mei Medical Center, Tainan 710, Taiwan; a108n2@mail.chimei.org.tw; 6 Institute of Biomedical Science, National Sun Yat-Sen University, Kaohsiung 804, Taiwan; 7 Department of Radiation Oncology, Chi Mei Medical Center, Tainan 710, Taiwan; 970816@mail.chimei.org.tw; 8 Department of Pharmacy, Chia-Nan University of Pharmacy and Science, Tainan 717, Taiwan; 9 Department of Internal Medicine, Chi Mei Medical Center, Tainan 710, Taiwan; 970402@mail.chimei.org.tw; 10 Department of Radiation Oncology, Chi Mei Medical Center, Liouying 736, Taiwan

**Keywords:** nasopharyngeal carcinoma, advanced nodal status, *SPIN4*, tight junction, prognostic biomarker

## Abstract

Nasopharyngeal carcinoma (NPC), characterized by the infiltration of lymphocytes, is a malignancy derived from the epithelium of the nasopharynx. Despite its sensitivity to radiation and chemotherapy, NPC has a high propensity for recurrence and metastasis. Although lymph node levels have been indicated as an independent prognostic factor for NPC, there has been no precise prognostic biomarker to predict clinical outcomes for NPC before advanced disease. In the present study, we surveyed differentially expressed genes in NPC via the next-generation sequencing (NGS)-based Oncomine database and identified the spindlin family member 4 (*SPIN4*) gene as the most relevant to advanced nodal status. We collected 124 tumor samples from NPC patients receiving biopsy, and the expression level of SPIN4 was evaluated by immunohistochemistry. The results showed that tumors with high SPIN4 expression were significantly correlated with advanced nodal status (*p* < 0.001) and advanced AJCC stages (*p* < 0.001). High SPIN4 expression in tumor samples was an unfavorable prognostic factor for all three endpoints at the univariate level: disease-specific survival (DSS), distal metastasis-free survival (DMeFS), and local recurrence-free survival (LRFS) (all *p* < 0.05). High SPIN4 expression remained independently prognostic of worse DMeFS (*p* = 0.049) at the multivariate level. Using bioinformatics analysis, we further found that high *SPIN4* level may link tight junctions to cancer cell survival. Collectively, these results imply that high SPIN4 expression is linked to an aggressive clinical course, including advanced nodal status and poor survival in NPC patients, emphasizing the promising prognostic utility of SPIN4 expression.

## 1. Introduction

Nasopharyngeal carcinoma (NPC) is a malignancy that is derived from the epithelium of the nasopharynx and has distinctive lymphoepithelial-like histological features. Generally, it occurs specifically in Southeast Asia, including Taiwan [1], and shows a male predominance in incidence, with a male to female ratio of 3:1. Based on World Health Organization (WHO) criteria, NPC is categorized into several histological subtypes: nonkeratinizing undifferentiated carcinoma, nonkeratinizing differentiated carcinoma, and keratinizing squamous cell carcinoma [2]. In endemic areas, NPC is mostly nonkeratinizing (differentiated or undifferentiated), which is strongly correlated with Epstein–Barr virus (EBV) infection [3]. Although EBV maintains lifelong latency in hosts, only a limited number of individuals infected with EBV develop cancer. This implies that other risk factors, such as genetic susceptibility and environmental exposures, are more likely to predispose individuals to develop this malignancy [4]. For NPC, patients with early-stage disease are sensitive to radiotherapy alone, and patients with locally advanced disease (stage II–IV disease) are responsive to radiation combined with cisplatin-based chemotherapy [5]. However, for patients with recurrent/metastatic disease, treatment options are still limited [5]. Fortunately, with the advancement of precision medicine, the identification of genetic biomarkers can improve treatment efficacy and risk stratification for patients with NPC.

Epigenetic alterations, including DNA methylation and histone acetylation and methylation, affect gene activity without changing the DNA sequence [6]. Generally, DNA methylation represses gene transcription, while histone acetylation promotes gene transcription [6]. Nonetheless, histone methylation can either activate or suppress the transcription of genes determined by which amino acids are methylated and how many methyl groups are attached [7]. Aberrant epigenetic and transcriptional regulation has been shown to be involved in cancer development, including NPC [4]. It has been reported that the activation of superenhancers, which can be revealed by the enhancer mark histone 3 lysine 27 acetylation (H3K27ac), is correlated with the oncogenic transcriptional network in NPC [8]. Moreover, the hypermethylation of HOP homeobox (*HOPX*), a tumor suppressor gene, has been suggested to be correlated with metastasis by activating *SNAIL* transcription in NPC [9]. Although NPC patients with the same disease stage undergo similar treatment, approximately 30% of them ultimately develop distant metastases, which is associated with disappointing treatment outcomes [10]. However, the correlations among aberrant epigenetic and transcriptional regulation, disease stages, and treatment efficacy in NPC remain poorly understood. Notably, there is no prospective genetic biomarker to precisely choose patients with a high risk of poor clinical outcomes.

Spindlin family member 4 (*SPIN4*), also known as TDRD28, is expressed mainly in the salivary gland and is also observed in the esophagus and lung (https://www.proteinatlas.org/ENSG00000186767-SPIN4/tissue, accessed on 27 July 2021). The *SPIN4* gene, which maps to chromosome Xq11.1 in humans, encodes a transcriptional coactivator harboring three Tudor domains [11]. Moreover, it can bind histone 3 lysine 4 trimethylation (H3K4me3), which is also correlated with the transcriptional activation of nearby genes [12]. The active marker H3K4me3 has been suggested to be enriched in NPC [13]. Additionally, in glioblastoma, the amplification of chromosomes Xq11.1–Xq11.2 has been linked to high *SPIN4* levels, which may activate the Wnt signaling pathway [14]. Targeting the Wnt/β-catenin pathway has also been reported to be a potential strategy for NPC therapy [15]. Nevertheless, the role of *SPIN4* in cancer still lacks investigation. In the present study, as *SPIN4* is expressed largely in the proximal digestive tract, we were interested in linking SPIN4 expression to clinical outcomes in our well-characterized NPC cohort.

## 2. Patients and Methods

### 2.1. Evaluation of the Gene Expression Profiles in Nasopharyngeal Carcinoma

To screen the potential genes that play a leading role in the oncogenesis of NPC, the next-generation sequencing (NGS)-based Oncomine database (https://www.oncomine.org/resource/login.html, accessed on 27 July 2021) was used to compare mRNA levels between nasopharyngeal tumors and normal tissues. We identified those with a *p*-value < 0.001 for further evaluation. Moreover, the top 20 differentially expressed genes were examined by comparing tumor samples with a nodal status beyond or identical to N1 (N1+) to those with negative nodal status (N0). Among these genes, *SPIN4*, with a prominent log2-transformed expression fold change, was selected for further analysis. To further predict the roles of *SPIN4* in NPC, the associations between the *SPIN4* mRNA level and its coexpressed genes in The Cancer Genome Atlas (TCGA) PanCancer Atlas database (n = 523) were examined utilizing the cBioPortal online platform (http://cbioportal.org, accessed on 27 July 2021). The top 200 genes with either positive associations or negative associations with *SPIN4* were further investigated using overrepresentation analysis in the PANTHER classification system (http://pantherdb.org, accessed on 27 July 2021) according to biological processes or cellular components and were rated by fold enrichment for functional annotation.

### 2.2. Patient Enrollment and Follow-Up

The Institutional Review Board of Chi Mei Medical Center approved the procurement of NPC tissues for this study (10501006). A total of 146 formalin-fixed paraffin-embedded (FFPE) tissue blocks of NPC patients who underwent biopsy between 1993 and 2002 were retrieved from our biobank. Among these patients, 10 with systemic disease and 12 who did not complete a standard course of therapy and/or were lost to tracking were excluded. The remaining 124 patients without distant metastasis at primary diagnosis were included in this retrospective study. All 124 patients received a daily dose of 180 to 200 cGy and 5 fractions weekly to accomplish a total dose of at least 7000 cGy in accordance with the published protocol [16]. The method of undergoing radiotherapy was generally consistent in this study. Generally, no less than three cycles of cisplatin-based chemotherapy were given for those with stage II–IV disease. Nevertheless, 1 patient with stage II disease and 4 patients with stage IV disease did not undergo chemotherapy but only radiotherapy owing to their poor general conditions. In addition, 7 patients were unavailable to acquire instant image assessments after therapy as a baseline to evaluate the response to treatment. Of these 124 patients, there were 7 partial and 110 complete responders based on a previously reported study modified from the WHO criteria [17]. In sum, 114 patients were routinely tracked after receiving therapy until death or their last follow-up, with a mean follow-up duration of 67 months (ranging from 3 to 141).

### 2.3. Histopathological and Immunohistochemical Appraisals

To acquire more objective results, two expert pathologists (Wan-Shan Li and Chien-Feng Li), who were blinded to the clinical and follow-up information of the patients, evaluated the histological subtypes in accordance with the WHO classification criteria. Tumor staging was assessed based on the 7th American Joint Committee on Cancer (AJCC) TNM staging system. The expression of EBV-encoded small RNA (EBER), which is indicative of EBV infection, was detected by in situ hybridization (ISH) based on our previous study [18]. Immunohistochemical staining was conducted based on our previous research [19], and staining was conducted with an anti-SPIN4 antibody (ab197353) (Abcam, Cambridge, MA, USA). The H-score was used to quantify SPIN4 immunoreactivity and was determined with the following equation: H-score = Σ*Pi* (*i* + 1), where *i* is the intensity of stained tumor cells (0 to 3+) and *Pi* is the percentage of staining for each intensity, ranging from 0% to 100% [20]. Tumors with H-scores beyond the median of all scored cases were regarded as having high SPIN4 expression.

### 2.4. Statistical Analysis

The χ^2^ test was used to evaluate the relationships between clinicopathological variables and SPIN4 expression status. The Kaplan–Meier method was utilized for survival analysis, and the log-rank test was performed to determine the interval from the starting date of radiotherapy to the event of interest. Variables with prognostic significance at the univariate level were incorporated into the Cox multivariate regression analysis to identify independent prognostic factors. The statistical analyses were performed in SPSS software version 22.0 (IBM Corporation, Armonk, NY, USA), and two-tailed tests with a *p*-value < 0.05 were considered statistically significant.

## 3. Results

### 3.1. SPIN4 Is Identified as the Most Significantly Differentially Expressed Gene in Relation to Advanced Nodal Status

**Figure 1:**
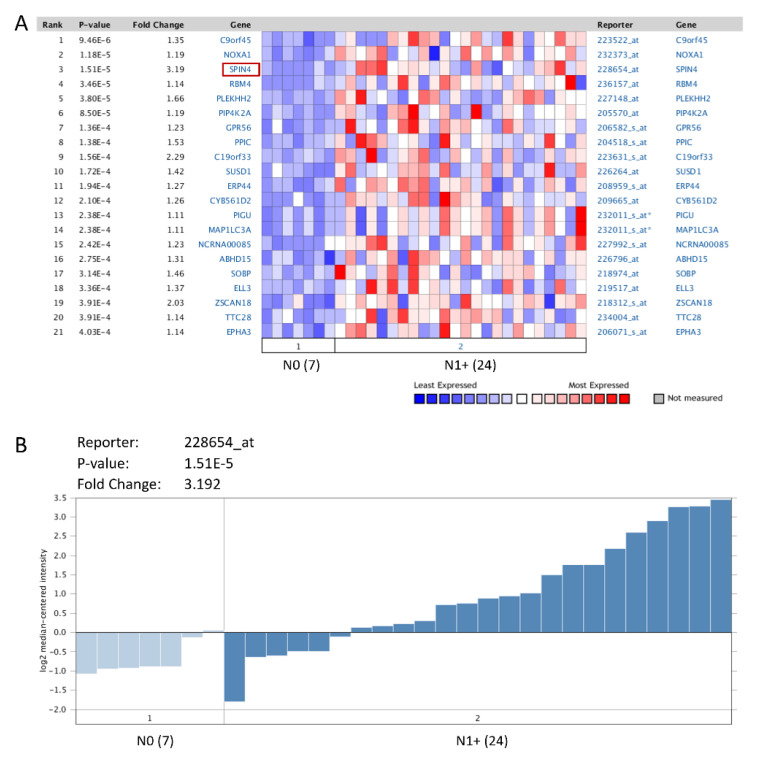
NGS data mining of genes associated with advanced nodal status in NPC. (**A**) The top 20 differentially expressed genes were examined by comparing tumor samples with a nodal status beyond or identical to N1 (N1+) to those with negative nodal status (N0). (**B**) Among these genes, *SPIN4*, with a prominent log2-transformed expression fold change, was selected for further analysis.

To investigate which genes play a leading role in the oncogenesis of NPC, the NGS-based Oncomine database was surveyed to identify prospective biomarkers. The statistical significance of each transcript was analyzed by comparing nasopharyngeal tumors to normal tissues, and those transcripts with a *p*-value < 0.001 were identified for further evaluation. As lymph node levels have been indicated as an independent prognostic factor for NPC [21], the top 20 differentially expressed genes were further analyzed by comparing tumor samples with a nodal status beyond or identical to N1 (N1+, n = 24) to those with negative nodal status (N0, n = 7) (Table 1 and Figure 1A). Among these genes, *SPIN4*, with a prominent log2-transformed expression fold change (log2 ratio = 3.1916), is expressed specifically in the salivary gland and was selected for further validation (Figure 1B).

### 3.2. Clinicopathological Characteristics of Nasopharyngeal Carcinoma Patients in Our Cohort

We collected 124 tumor samples from nasopharyngeal carcinoma patients undergoing biopsy from 1993 to 2002; most patients were male (n = 95, 76.6%) and less than 60 years old (n = 98, 79%) (Table 2). In terms of primary tumor status, 80 (64.5%) patients were classified as early status (T1–T2), and 44 (35.5%) patients were classified as advanced status (T3–T4). With respect to initial nodal status, 56 (45.2%) patients were categorized as early status (N0–N1), and 68 (54.8%) patients were categorized as advanced status (N2–N3). Moreover, stage I–II disease was observed in 38 (30.6%) patients, and stage III–IV disease was detected in 86 (69.4%) patients. In addition, EBER, which is indicative of EBV infection, was expressed in 123 (99.2%) patients.

### 3.3. SPIN4 Immunoexpression and Its Associations with Clinicopathological Variables

**Figure 2:**
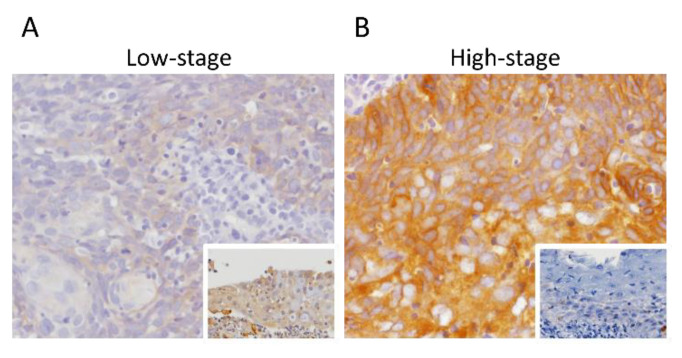
Immunohistochemical detection of SPIN4. Representative images of nasopharyngeal carcinoma exhibiting lower SPIN4 immunoexpression (brown–orange) among (**A**) NPC patients with low-stage compared to that among (**B**) NPC patients with high-stage. Inset: adjacent non-neoplastic mucosa.

To analyze the associations between SPIN4 immunoexpression and its clinical relevance in nasopharyngeal carcinoma, immunohistochemical staining was performed (Table 2). High SPIN4 expression was significantly correlated with advanced nodal status (*p* < 0.001) and advanced AJCC stages (*p* < 0.001), whereas it was not associated with histological subtypes or EBER expression. As shown in Figure 2A,B, SPIN4 immunoreactivity in NPC specimens with high-stage was significantly higher than that in NPC specimens with low-stage.

### 3.4. Survival and Prognostic Impact of SPIN4 Expression in Nasopharyngeal Carcinoma

**Figure 3:**
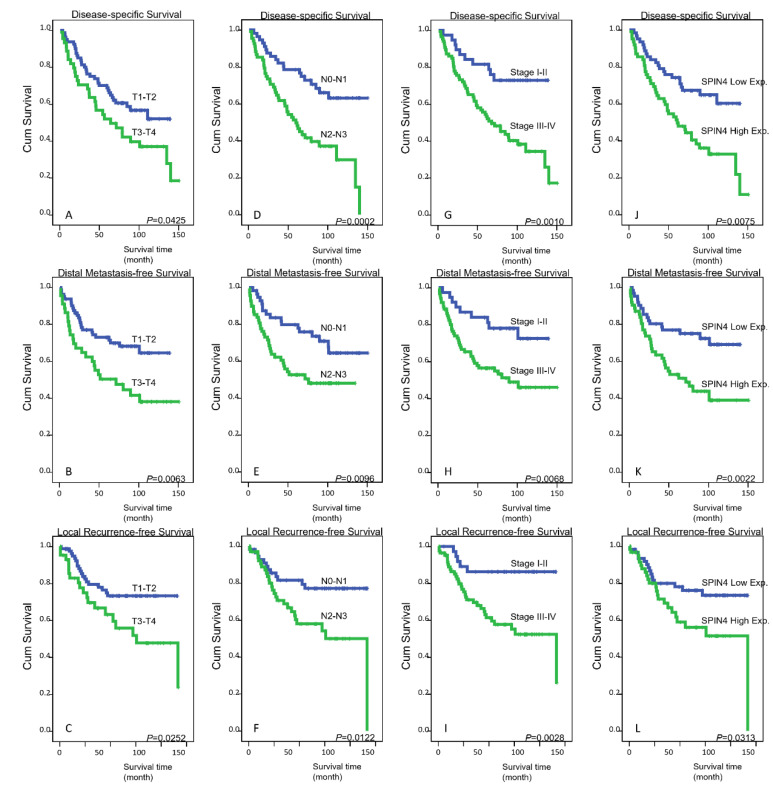
Survival analysis. Kaplan–Meier curves were plotted and revealed that (**A–C**) T3–T4 status, (**D**–**F**) N2–N3 status, and (**G**–**I**) AJCC stage III–IV tumors and (**J**–**L**) high SPIN4 expression in tumor specimens were all significantly correlated with poor DSS, DMeFS, and LRFS.

Patients with T3–T4 status, N2–N3 status, and AJCC stage III–IV disease at primary diagnosis were all markedly associated with three endpoints at the univariate level: disease-specific survival (DSS), distal metastasis-free survival (DMeFS), and local recurrence-free survival (LRFS) (all *p* < 0.05) (Table 3 and Figure 3A–I). In addition, high SPIN4 expression in tumor specimens was also unfavorably prognostic of all three endpoints (all *p* < 0.05) (Figure 3J–L) analyzed. Notably, at the multivariate level, AJCC stages III–IV were still markedly associated with worse DSS (*p* = 0.028) and LRFS (*p* = 0.019), and high SPIN4 expression remained an independent prognostic factor for inferior DMeFS (*p* = 0.049) (Table 4).

### 3.5. High SPIN4 Level May Link Tight Junctions to Cancer Cell Survival

To forecast the specific roles of *SPIN4* in NPC, a gene coexpression analysis was performed. Utilizing the TCGA database (n = 523), we estimated the top 200 transcripts that showed positive (Supplementary Table S1) or negative (Supplementary Table S2) correlations with *SPIN4*. In the context of biological processes and cellular components, the most significant terms correlated with *SPIN4* upregulation were establishment of endothelial intestinal barrier (GO: 0090557, fold enrichment: 41.86, *p* = 4.98 × 10^−7^) and tight junction (GO: 0070160, fold enrichment: 6.28, *p* = 6.22 × 10^−5^), respectively (Supplementary Figure S1A,B), utilizing the PANTHER classification system. Moreover, we recognized that the tight junction protein 1 (*TJP1*) (Spearman’s correlation: 0.382) and *TJP2* (Spearman’s correlation: 0.394) (Supplementary Figure S2A,B) genes are both involved in the above two terms, implying that *SPIN4* is functionally associated with the protective role of tight junctions. Additionally, TJP1 and TJP2 have also been linked to cell proliferation and survival [22].

## 4. Discussion

Despite the fact that the correlation of EBV infection with NPC is well-recognized, only a limited number of individuals infected with EBV develop NPC, suggesting that other risk factors such as genetic changes may play a more important role in NPC development [4]. In this study, to discover the potential genes that play a leading role in the oncogenesis of NPC, we performed NGS data mining and identified the *SPIN4* gene as the most relevant to advanced nodal status, which serves as a useful prognostic factor for NPC. Immunohistochemical staining revealed that high SPIN4 expression is linked to an aggressive clinical course, including advanced nodal status and inferior survival in our well-characterized NPC cohort, highlighting the promising prognostic utility of SPIN4 expression.

Extensive histone modifications have been observed in EBV-infected NPC cells. It has been demonstrated that H3K27ac-driven enhancer dysfunction correlates with a worse prognosis in NPC [23]. H3K27me3 modification has also been used to predict the chemoradiotherapy response and survival for NPC patients [24]. Furthermore, EBV infection is associated with aberrant histone bivalent switches by enhancing the transcription-suppressive mark (H3K27me3) and reducing the transcriptional activation mark (H3K4me3), and downregulates the DNA damage repair gene [25]. However, our data revealed that EBV significantly correlates with neither SPIN4 expression nor clinical outcomes. Interestingly, SPIN4 exhibits H3K4me3-binding activity [12] and functions as a transcriptional coactivator. Using bioinformatics analysis, we further found that the tight junction proteins *TJP1* and *TJP2* show significant positive correlations with *SPIN4* and are linked to cancer cell survival [22]. In addition, it has been reported that H3K4me3 can activate *TJP1* transcription [26]. These suggest that SPIN4 may increase *TJP1* transcripts through H3K4me3-mediated transcriptional activation. Collectively, SPIN4 may promote cancer cell survival in an EBV-independent manner and correlate with poor patient survival, further reflecting the intricate regulation of NPC.

In general, transcription factors bind enhancers and recruit coactivators and RNA polymerase II, which generates a general transcription machinery to regulate gene expression [27]. Several transcription factors have been demonstrated to be oncogenic and incorporate the general transcription machinery to support the oncogenic state, but direct inhibition of transcription factors remains difficult. As a transcriptional coregulator, a coactivator can bind to a transcription factor to increase the rate of gene transcription. Recently, multiple approaches, including inhibition of transcription factor-coactivator interactions, have been developed to target transcription factors [28]. Additionally, the dynamic character of DNA methylation makes it reversible and druggable and represents a potential prognostic factor for cancers [29]. Since SPIN4 acts as a transcriptional coactivator and can bind H3K4me3 to promote gene transcription, targeting SPIN4 and H3K4me3 is feasible and represents a promising therapeutic strategy for NPC patients.

Since *TJP1* and *TJP2* were significantly correlated with *SPIN4* upregulation, we wondered how these tight junction proteins participate in NPC progression. Tight junctions coordinate with adherens junctions and focal adhesions to form cell adhesion complexes, and such interconnected networks can guide diverse cell functions [22]. Regulated by cell adhesion to the extracellular matrix (ECM), cellular stiffness can enhance resistance to radiation and chemotherapy [30]. Adherens junctions and focal adhesions have been indicated to be functional biomarkers in NPC [31]. Moreover, focal adhesion kinase (FAK) has been suggested to maintain the intestinal epithelial barrier through the assembly of tight junction proteins in colorectal cancer cells [32]. In addition, cancer stem cells (CSCs) are awakened by radiation to induce tumor recurrence and metastasis in oral cancer patients [33]. Tight junction proteins, including TJP1, have been suggested to maintain the intercellular configuration of neural stem cells [34]. In addition to activating *TJP1* transcription, [26], H3K4me3 has been suggested to play a critical role in the genetic regulation of pluripotency [35]. However, the correlations among the expression of SPIN4 and tight junction proteins, radioresistance, and stem cell maintenance deserve further investigation.

Despite its sensitivity to radiation and chemotherapy, NPC easily develops recurrence and metastasis. NPC is characterized by the infiltration of lymphocytes and positive programmed death-ligand 1 (PD-L1) expression in tumor cells, which makes it an attractive target for immune checkpoint inhibitors (ICIs). Although the phase I–II trial outcomes of immunotherapy were encouraging in NPC patients with recurrent or metastatic disease, there has been no treatment paradigm applying ICIs to this malignancy [36]. This suggests that unknown factors may affect immunotherapy efficacy. Additionally, a valuable predictive biomarker for immunotherapy in NPC is still lacking. Through comprehensive single-cell sequencing, it has been revealed that myeloid-derived suppressor cells (MDSCs) and tumor-associated macrophages (TAMs) serve as a connecting bridge to facilitate the immunosuppressive tumor microenvironment (TME) in NPC [37]. In the TME, tumor cells and nonmalignant cells such as immune cells interact with each other and are closely linked. It has been reported that tight junctions are crucial to maintain this tumor cell–nonmalignant cell interaction, which may sustain the immunosuppressive TME [38]. Furthermore, since the immunosuppressive TME can affect immunotherapy efficacy, targeting the TME alone or combined with ICIs may benefit cancer patients [39]. Accordingly, whether SPIN4 and tight junction proteins function as predictive biomarkers and therapeutic targets for improving immunotherapy in NPC patients warrants further investigation.

Since copy number alteration may affect tumor development through changes in gene expression, the amplification of chromosomes Xq11.1–Xq11.2 has been linked to high *SPIN4* levels in glioblastoma [14]. In addition, the gain of chromosome X has been connected to neoplastic transformation, including prostate cancer [40], breast cancer [41], and renal cell carcinoma [42]. We also found that high SPIN4 expression is an unfavorable prognostic factor for renal cancer using the Human Protein Atlas database (https://www.proteinatlas.org/ENSG00000186767-SPIN4/pathology/renal+cancer, accessed on 27 July 2021). Furthermore, copy number variation has been suggested to be correlated with chromosome X-linked mental retardation, with an excess of affected males [43]. Transcription factors are regarded as a trigger of cancer initiation. Accordingly, as an X-linked transcriptional coactivator, SPIN4 overexpression may in part provide an explanation of why males dominate in the incidence of NPC.

## 5. Conclusions

As lymph node levels have been indicated as an independent prognostic factor for NPC, we performed NGS data mining related to advanced nodal status and analyzed the relationships between SPIN4 immunoexpression and its clinical relevance in our well-characterized NPC cohort. We demonstrated that high SPIN4 expression is connected to an aggressive clinical course, including advanced nodal status, and functions as an independent prognostic biomarker for inferior DMeFS in NPC patients.

## Figures and Tables

**Table 1:** The top 20 overexpressed genes in nasopharyngeal carcinoma associated with advanced nodal status.

Gene Rank	Gene Name	Gene Symbol	Reporter ID	*p*-Value	*q*-Value	Fold Change
1	chromosome 9 open reading frame 45	*C9orf45*	223522_at	9.46E-06	0.516966	1.350599
2	NADPH oxidase activator 1	*NOXA1*	232373_at	1.18E-05	0.321552	1.190006
3	spindlin family, member 4	*SPIN4*	228654_at	1.51E-05	0.274995	3.191617
4	RNA binding motif protein 4	*RBM4*	236157_at	3.46E-05	0.473519	1.144074
5	pleckstrin homology domain containing, family H (with MyTH4 domain) member 2	*PLEKHH2*	227148_at	3.80E-05	0.415277	1.659764
6	phosphatidylinositol-5-phosphate 4-kinase, type II, alpha	*PIP4K2A*	205570_at	8.50E-05	0.774191	1.18786
7	G protein-coupled receptor 56	*GPR56*	206582_s_at	1.36E-04	0.744635	1.227697
8	peptidylprolyl isomerase C (cyclophilin C)	*PPIC*	204518_s_at	1.38E-04	0.685162	1.528623
9	chromosome 19 open reading frame 33	*C19orf33*	223631_s_at	1.56E-04	0.710361	2.287249
10	sushi domain containing 1	*SUSD1*	226264_at	1.72E-04	0.721422	1.420516
11	endoplasmic reticulum protein 44	*ERP44*	208959_s_at	1.94E-04	0.663589	1.266438
12	cytochrome b-561 domain containing 2	*CYB561D2*	209665_at	2.10E-04	0.674904	1.264451
13	phosphatidylinositol glycan anchor biosynthesis, class U	*PIGU*	232011_s_at	2.38E-04	0.723024	1.110637
14	microtubule-associated protein 1 light chain 3 alpha	*MAP1LC3A*	232011_s_at	2.38E-04	0.723024	1.110637
15	non-protein coding RNA 85	*NCRNA00085*	227992_s_at	2.42E-04	0.695161	1.233108
16	abhydrolase domain containing 15	*ABHD15*	226796_at	2.75E-04	0.750663	1.305885
17	sine oculis binding protein homolog (Drosophila)	*SOBP*	218974_at	3.14E-04	0.781121	1.46335
18	elongation factor RNA polymerase II-like 3	*ELL3*	219517_at	3.36E-04	0.797915	1.369774
19	zinc finger and SCAN domain containing 18	*ZSCAN18*	218312_s_at	3.91E-04	0.854255	2.034354
20	tetratricopeptide repeat domain 28	*TTC28*	234004_at	3.91E-04	0.82273	1.14447

**Table 2:** Associations between SPIN4 expression and other important clinicopathological variables.

Parameters	Category	SPIN4 Exp.		*p*-Value
		Low	High	
Gender	Male	46	49	0.524
	Female	16	13	
Age (years)	<60 years	46	52	0.186
	≥60 years	16	14	
Primary tumor (T)	T1–T2	44	36	0.133
	T3–T4	18	26	
Nodal status (N)	N0–N1	45	11	<0.001 *
	N2–N3	17	51	
Stage	I–II	32	6	<0.001 *
	III–IV	30	56	
Histological grade	Keratinizing	3	2	0.814
	Nonkeratinizing	28	26	
	Undifferentiated	31	34	
EBER	Negative	0	1	0.315
	Positive	62	61	

* statistically significant.

**Table 3:** Univariate log-rank analyses.

Parameters	Category	No. of Case	DSS		DMeFS		LRFS	
			No. of Event	*p*-Value	No. of Event	*p*-Value	No. of Event	*p*-Value
Gender	Male	95	48	0.8104	39	0.5524	31	0.3313
	Female	29	15		11		7	
Age (years)	<60 years	98	52	0.5857	42	0.4146	30	0.8804
	≥60 years	26	11		8		8	
Primary tumor (T)	T1–T2	80	34	0.0425 *	25	0.0063 *	19	0.0252 *
	T3–T4	44	29		25		19	
Nodal status (N)	N0–N1	56	19	0.0002 *	17	0.0096 *	12	0.0122 *
	N2–N3	68	44		33		26	
Stage	I–II	38	10	0.0010 *	9	0.0068 *	5	0.0028 *
	III–IV	86	53		41		33	
Histological grade	Keratinizing/non-keratinizing	47	24	0.4120	17	0.2155	16	0.9323
	Undifferentiated	77	39		33		22	
EBER	Negative	1	1	0.0567	1	0.0923	0	0.7314
	Positive	123	62		49		38	
*SPIN4* exp.	Low exp. (H-score < median)	66	25	0.0075 *	17	0.0022 *	15	0.0313 *
	High exp. (H-score ≥ median)	58	34		33		23	

DSS, disease-specific survival; DMeFS, distal metastasis-free survival; LRFS, local recurrence-free survival; *, statistically significant.

**Table 4:** Multivariate survival analyses.

Parameter	Category	DSS			DMeFS			LRFS		
		H.R	95% CI	*p*-Value	H.R	95% CI	*p*-Value	H.R	95% CI	*p*-Value
Stage	I–II	1	-	0.028 *	1	-	0.104	1	-	0.019 *
	III–IV	2.275	1.029–4.740		1.922	0.874–4.228		3.288	1.215−8.901	
*SPIN4* exp.	Low Exp.	1	-	0.056	1	-	0.049 *	1	-	0.345
	High Exp.	1.731	0.986–3.040		1.905	1.003–3.617		1.394	0.700–2.776	

DSS, disease-specific survival; DMeFS, distal metastasis-free survival; LRFS, local recurrence-free survival; *, statistically significant.

## Data Availability

The Oncomine database (https://www.oncomine.org/resource/login.html, accessed on 27 July 2021) is a cancer-related gene database that contains Gene Expression Omnibus (GEO), TCGA, microarray data, and published research data. The database contains 715 gene expression datasets, which comprise 86,733 tumors and normal tissues.

## Supplementary Materials

The following are available online at https://www.mdpi.com/article/10.3390/life11090912/s1: Figure S1. The top 10 biological process or cellular component terms enriched in *SPIN4* upregulation. The genes that were coexpressed with *SPIN4* in NPC from the TCGA database (n = 523) were analyzed using the cBioPortal online platform. The genes (top 200 transcripts) with positive associations were further analyzed using the PANTHER classification system in accordance with (A) biological processes or (B) cellular components and were rated by fold enrichment for functional annotation; Figure S2. Associations among the expression levels of *SPIN4,* *TJP1*, and *TJP2*. The data were exported from the TCGA database (n = 523) using the cBioPortal online platform; Table S1. The top 200 genes positively correlated with *SPIN4*; Table S2. The top 200 genes negatively correlated with *SPIN4*.
