# Supplementary material for: High SPIN4 Expression Is Linked to Advanced Nodal Status and Inferior Prognosis in Nasopharyngeal Carcinoma Patients
Source: Life (Basel). 2021 Sep 1;11(9):912. doi: 10.3390/life11090912 (PMC8470428; doi:10.3390/life11090912)
Supplement: Supplementary file 1 [file life-11-00912-s001.zip › life-1337082-supplementary.pdf]

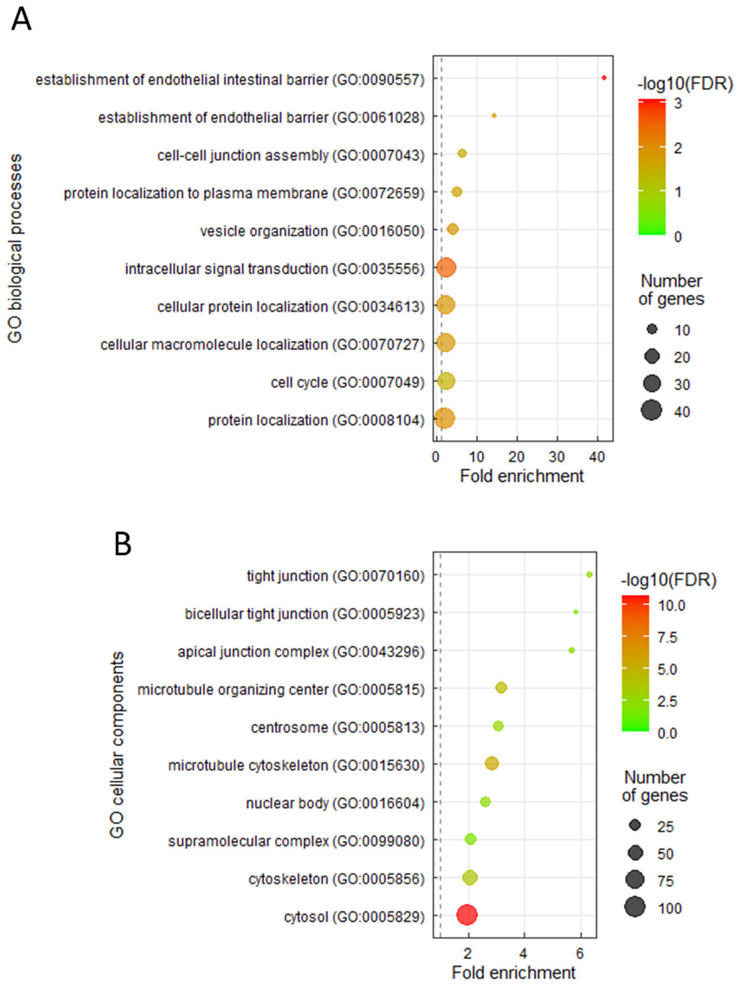

Figure S1. The top 10 biological process or cellular component terms enriched in *SPIN4* upregulation. The genes that were coexpressed with *SPIN4* in NPC from the TCGA database (n = 523) were analyzed using the cBioPortal online platform. The genes (top 200 transcripts) with positive associations were further analyzed using the PANTHER classification system in accordance with (**A**) biological processes or (**B**) cellular components and were rated by *fold enrichment* for functional annotation.

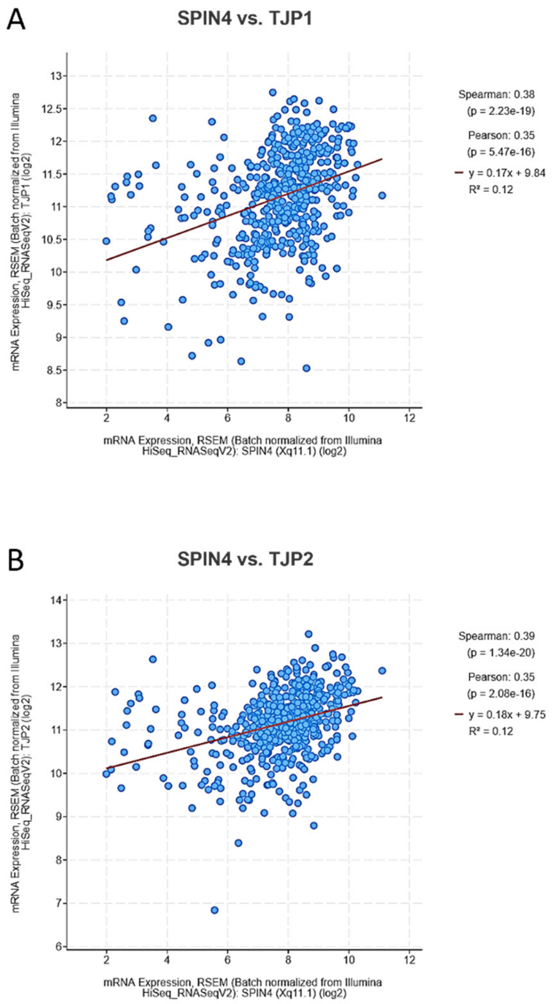

Figure S2. Associations among the expression levels of *SPIN4*, *TJP1*, and *TJP2*. *The data were exported from* the TCGA database (n = 523) using the cBioPortal online platform.

Table S1. The top 200 genes positively correlated with *SPIN4*.

Correlated Gene	Cytoband	Spearman's Correlation	*p*-Value	q-Value
**RAB27B**	18q21.2	0.474	3.05E-30	**6.11E-26**
**IRAK2**	3p25.3	0.468	2.08E-29	**2.08E-25**
**PDCD6IP**	3p22.3	0.442	5.43E-26	**2.18E-22**
**FCHO2**	5q13.2	0.438	1.32E-25	**3.78E-22**
**TAB3**	Xp21.2	0.433	6.67E-25	**1.34E-21**
**EPS15**	1p32.3	0.425	5.24E-24	**9.55E-21**
**ADGRG6**	6q24.2	0.423	9.65E-24	**1.61E-20**
**PKN2**	1p22.2	0.421	1.37E-23	**2.11E-20**
**CLASP2**	3p22.3	0.42	1.86E-23	**2.67E-20**
**MIER1**	1p31.3	0.419	2.90E-23	**3.75E-20**
**SLMAP**	3p14.3	0.418	3.00E-23	**3.75E-20**
**GNG12**	1p31.3	0.417	4.84E-23	**5.71E-20**
**PTAFR**	1p35.3	0.415	6.73E-23	**7.49E-20**
**ITSN2**	2p23.3	0.414	1.02E-22	**1.03E-19**
**SLC25A24**	1p13.3	0.412	1.48E-22	**1.41E-19**
**ANKRD13C**	1p31.1	0.41	2.65E-22	**2.31E-19**
**CRYBG1**	6q21	0.409	3.09E-22	**2.48E-19**

**TMOD3**	15q21.2	0.409	3.23E-22	**2.49E-19**
**MORC3**	21q22.12	0.406	6.97E-22	**4.66E-19**
**TRIM33**	1p13.2	0.403	1.51E-21	**9.15E-19**
**SLK**	10q24.33-q25.1	0.402	1.97E-21	**1.16E-18**
**ERI1**	8p23.1	0.399	4.39E-21	**2.44E-18**
**BMP2K**	4q21.21	0.398	5.11E-21	**2.69E-18**
**USP53**	4q26	0.397	6.52E-21	**3.35E-18**
**OXSR1**	3p22.2	0.397	7.56E-21	**3.70E-18**
**PDS5A**	4p14	0.396	9.40E-21	**4.49E-18**
**TJP2**	9q21.11	0.394	1.34E-20	**6.09E-18**
**GSTCD**	4q24	0.394	1.38E-20	**6.14E-18**
**PI4K2B**	4p15.2	0.393	1.62E-20	**7.05E-18**
**UGCG**	9q31.3	0.392	2.30E-20	**9.59E-18**
**RAP2C**	Xq26.2	0.39	3.53E-20	**1.39E-17**
**AMPD3**	11p15.4	0.39	3.76E-20	**1.45E-17**
**TAOK3**	12q24.23	0.389	4.33E-20	**1.64E-17**
**MAST4**	5q12.3	0.386	9.79E-20	**3.38E-17**
**ABCD3**	1p21.3	0.385	1.26E-19	**4.00E-17**
**CLOCK**	4q12	0.384	1.58E-19	**4.96E-17**
**CHM**	Xq21.2	0.383	1.96E-19	**6.05E-17**
**TJP1**	15q13.1	0.382	2.23E-19	**6.67E-17**
**OTUD4**	4q31.21	0.382	2.52E-19	**7.42E-17**
**KBTBD8**	3p14.1	0.381	2.98E-19	**8.18E-17**
**CCDC186**	10q25.3	0.381	2.98E-19	**8.18E-17**
**BACH1**	21q21.3	0.38	3.79E-19	**1.03E-16**
**DDX3X**	Xp11.4	0.379	4.61E-19	**1.22E-16**
**DHX15**	4p15.2	0.379	4.87E-19	**1.27E-16**
**MTF1**	1p34.3	0.379	5.28E-19	**1.36E-16**
**ZFYVE16**	5q14.1	0.378	5.99E-19	**1.52E-16**
**LTN1**	21q21.3	0.378	6.43E-19	**1.59E-16**
**EEA1**	12q22	0.377	7.22E-19	**1.76E-16**
**ARHGEF3**	3p14.3	0.377	7.92E-19	**1.89E-16**
**NRAS**	1p13.2	0.375	1.24E-18	**2.77E-16**
**FAM199X**	Xq22.2	0.373	1.96E-18	**4.27E-16**
**NIPAL1**	4p12	0.372	2.67E-18	**5.70E-16**
**RREB1**	6p24.3	0.369	4.24E-18	**8.34E-16**
**MBNL3**	Xq26.2	0.369	4.40E-18	**8.52E-16**
**FRYL**	4p11	0.368	6.37E-18	**1.20E-15**
**RBM41**	Xq22.3	0.368	6.39E-18	**1.20E-15**
**LIG4**	13q33.3	0.367	6.58E-18	**1.22E-15**
**FAM126B**	2q33.1	0.366	8.27E-18	**1.52E-15**
**VPS4B**	18q21.33	0.366	8.77E-18	**1.58E-15**
**USP33**	1p31.1	0.365	1.05E-17	**1.88E-15**
**MED14**	Xp11.4	0.365	1.07E-17	**1.89E-15**
**CPEB3**	10q23.32	0.365	1.18E-17	**2.07E-15**
**PGM2**	4p14	0.364	1.40E-17	**2.42E-15**
**SEC24B**	4q25	0.364	1.43E-17	**2.46E-15**
**FER**	5q21.3	0.364	1.49E-17	**2.53E-15**
**EHF**	11p13	0.363	1.65E-17	**2.75E-15**
**PTPN13**	4q21.3	0.363	1.66E-17	**2.75E-15**
**EPG5**	18q12.3-q21.1	0.362	1.98E-17	**3.21E-15**

**CRLF3**	17q11.2	0.362	2.07E-17	**3.27E-15**
**WDR44**	Xq24	0.361	2.91E-17	**4.33E-15**
**GMEB1**	1p35.3	0.36	3.10E-17	**4.46E-15**
**PKP2**	12p11.21	0.36	3.18E-17	**4.56E-15**
**HIPK1**	1p13.2	0.36	3.31E-17	**4.70E-15**
**ITPR2**	12p11.23	0.36	3.40E-17	**4.80E-15**
**RAD18**	3p25.3	0.36	3.59E-17	**5.00E-15**
**CD2AP**	6p12.3	0.359	4.03E-17	**5.53E-15**
**CNOT6**	5q35.3	0.359	4.45E-17	**6.03E-15**
**MPP5**	14q23.3	0.359	4.59E-17	**6.14E-15**
**PRKAR2A**	3p21.31	0.358	4.75E-17	**6.31E-15**
**GCOM1**	15q21.3	0.358	5.10E-17	**6.73E-15**
**NCOA4**	10q11.22	0.358	5.38E-17	**7.01E-15**
**MBP**	18q23	0.357	6.60E-17	**8.38E-15**
**DMXL1**	5q23.1	0.357	6.70E-17	**8.45E-15**
**STK4**	20q13.12	0.356	7.46E-17	**9.34E-15**
**OSBPL8**	12q21.2	0.356	7.50E-17	**9.34E-15**
**STRN**	2p22.2	0.356	7.71E-17	**9.55E-15**
**EAF1**	3p25.1	0.356	8.03E-17	**9.88E-15**
**C18ORF25**	18q21.1	0.356	8.47E-17	**1.03E-14**
**TASOR**	3p14.3	0.355	8.95E-17	**1.08E-14**
**SERPINB2**	18q21.33-q22.1	0.355	9.64E-17	**1.14E-14**
**USP24**	1p32.3	0.354	1.09E-16	**1.27E-14**
**ATXN7**	3p14.1	0.353	1.39E-16	**1.55E-14**
**SGMS2**	4q25	0.353	1.44E-16	**1.59E-14**
**ELOVL7**	5q12.1	0.353	1.50E-16	**1.66E-14**
**TNKS2**	10q23.32	0.353	1.54E-16	**1.69E-14**
**MINDY2**	15q21.3-q22.1	0.352	1.70E-16	**1.83E-14**
**CMTR2**	16q22.2	0.352	1.89E-16	**2.02E-14**
**KIF20B**	10q23.31	0.351	2.03E-16	**2.17E-14**
**RASSF3**	12q14.2	0.351	2.17E-16	**2.30E-14**
**USP38**	4q31.21	0.351	2.48E-16	**2.58E-14**
**HNRNPF**	10q11.21	0.35	2.53E-16	**2.62E-14**
**KAT2B**	3p24.3	0.349	3.12E-16	**3.13E-14**
**MTM1**	Xq28	0.349	3.17E-16	**3.17E-14**
**MBLAC2**	5q14.3	0.349	3.67E-16	**3.57E-14**
**NFKB1**	4q24	0.348	3.84E-16	**3.68E-14**
**LRRC40**	1p31.1	0.348	3.84E-16	**3.68E-14**
**NBPF10**	1q21.1	0.348	4.03E-16	**3.84E-14**
**PHACTR2**	6q24.2	0.348	4.08E-16	**3.84E-14**
**SERPINB8**	18q22.1	0.347	4.65E-16	**4.31E-14**
**GOLGA4**	3p22.2	0.347	4.93E-16	**4.53E-14**
**GPSM2**	1p13.3	0.346	6.24E-16	**5.63E-14**
**BDP1**	5q13.2	0.345	6.98E-16	**6.25E-14**
**DENND2C**	1p13.2	0.345	7.50E-16	**6.65E-14**
**SMC1A**	Xp11.22	0.345	7.84E-16	**6.89E-14**
**SLC28A3**	9q21.32-q21.33	0.345	8.02E-16	**7.02E-14**
**PRR11**	17q22	0.344	8.70E-16	**7.55E-14**
**HIVEP1**	6p24.1	0.344	8.84E-16	**7.64E-14**
**DENND4C**	9p22.1	0.344	9.82E-16	**8.45E-14**
**MYD88**	3p22.2	0.343	1.04E-15	**8.86E-14**

**DDI2**	1p36.21	0.343	1.04E-15	**8.86E-14**
**CARNMT1**	9q21.13	0.343	1.16E-15	**9.70E-14**
**LYSMD3**	5q14.3	0.343	1.16E-15	**9.70E-14**
**CTTNBP2NL**	1p13.2	0.343	1.19E-15	**9.98E-14**
**EYA3**	1p35.3	0.343	1.21E-15	**1.01E-13**
**POLR3A**	10q22.3	0.343	1.25E-15	**1.02E-13**
**PBRM1**	3p21.1	0.342	1.29E-15	**1.04E-13**
**DENND6A**	3p14.3	0.341	1.65E-15	**1.30E-13**
**C12ORF29**	12q21.32	0.341	1.67E-15	**1.31E-13**
**YTHDC2**	5q22.2	0.341	1.67E-15	**1.31E-13**
**RSC1A1**	1p36.21	0.34	1.92E-15	**1.49E-13**
**GORASP1**	3p22.2	0.339	2.39E-15	**1.82E-13**
**RASA1**	5q14.3	0.339	2.53E-15	**1.90E-13**
**FOXN2**	2p16.3	0.339	2.53E-15	**1.90E-13**
**DOCK9**	13q32.3	0.339	2.67E-15	**2.00E-13**
**VPS13A**	9q21.2	0.338	2.86E-15	**2.13E-13**
**STXBP3**	1p13.3	0.338	3.06E-15	**2.27E-13**
**MALT1**	18q21.32	0.338	3.09E-15	**2.29E-13**
**MICALCL**	11p15.3	0.338	3.12E-15	**2.30E-13**
**KIF2A**	5q12.1	0.338	3.21E-15	**2.35E-13**
**SYNJ1**	21q22.11	0.337	3.52E-15	**2.58E-13**
**PTBP3**	9q32	0.337	3.99E-15	**2.86E-13**
**AQR**	15q14	0.337	4.07E-15	**2.90E-13**
**ASCC3**	6q16.3	0.337	4.15E-15	**2.93E-13**
**RNF144B**	6p22.3	0.336	4.50E-15	**3.13E-13**
**TTC37**	5q15	0.336	4.77E-15	**3.29E-13**
**PATL1**	11q12.1	0.336	5.10E-15	**3.50E-13**
**GABPA**	21q21.3	0.335	5.66E-15	**3.86E-13**
**METTL14**	4q26	0.335	5.90E-15	**4.00E-13**
**APAF1**	12q23.1	0.335	5.90E-15	**4.00E-13**
**PDE12**	3p14.3	0.335	6.22E-15	**4.18E-13**
**SLC38A1**	12q13.11	0.334	6.91E-15	**4.60E-13**
**RP2**	Xp11.3	0.334	7.08E-15	**4.68E-13**
**ARAP2**	4p14	0.333	8.31E-15	**5.31E-13**
**DIP2A**	21q22.3	0.333	8.49E-15	**5.39E-13**
**FBXO3**	11p13	0.333	8.50E-15	**5.39E-13**
**HERC3**	4q22.1	0.333	8.99E-15	**5.69E-13**
**NDFIP2**	13q31.1	0.333	9.20E-15	**5.79E-13**
**CAB39**	2q37.1	0.331	1.18E-14	**7.35E-13**
**FMR1**	Xq27.3	0.331	1.20E-14	**7.46E-13**
**EPB41L5**	2q14.2	0.331	1.23E-14	**7.61E-13**
**EIF3A**	10q26.11	0.331	1.25E-14	**7.67E-13**
**RCAN3**	1p36.11	0.33	1.38E-14	**8.40E-13**
**MYO18A**	17q11.2	0.33	1.41E-14	**8.54E-13**
**CLSPN**	1p34.3	0.33	1.43E-14	**8.59E-13**
**NCOA2**	8q13.3	0.33	1.65E-14	**9.89E-13**
**SLC4A7**	3p24.1	0.329	1.70E-14	**1.01E-12**
**APPL1**	3p14.3	0.329	1.88E-14	**1.11E-12**
**HS3ST1**	4p15.33	0.329	1.88E-14	**1.11E-12**
**GAS2L3**	12q23.1	0.329	1.96E-14	**1.15E-12**
**BRWD3**	Xq21.1	0.328	2.25E-14	**1.31E-12**

**KIF13B**	8p12	0.328	2.35E-14	**1.36E-12**
**NEK7**	1q31.3	0.328	2.35E-14	**1.36E-12**
**RIC1**	9p24.1	0.327	2.57E-14	**1.48E-12**
**CCDC15**	11q24.2	0.327	2.57E-14	**1.48E-12**
**CREB1**	2q33.3	0.327	2.66E-14	**1.53E-12**
**ARNTL2**	12p11.23	0.327	2.74E-14	**1.56E-12**
**CAPZA1**	1p13.2	0.327	2.86E-14	**1.62E-12**
**WDR11**	10q26.12	0.326	2.96E-14	**1.68E-12**
**PPP4R2**	3p13	0.326	3.00E-14	**1.69E-12**
**RBM47**	4p14	0.326	3.01E-14	**1.69E-12**
**TMF1**	3p14.1	0.326	3.09E-14	**1.73E-12**
**FBXL3**	13q22.3	0.326	3.17E-14	**1.76E-12**
**RNF169**	11q13.4	0.326	3.26E-14	**1.81E-12**
**WAPL**	10q23.2	0.326	3.33E-14	**1.84E-12**
**RELCH**	18q21.33	0.325	3.60E-14	**1.97E-12**
**AP4E1**	15q21.2	0.325	3.70E-14	**2.01E-12**
**FBXW11**	5q35.1	0.325	3.78E-14	**2.05E-12**
**SLC25A43**	Xq24	0.325	3.86E-14	**2.09E-12**
**CHAMP1**	13q34	0.325	3.89E-14	**2.10E-12**
**TFDP1**	13q34	0.325	4.04E-14	**2.17E-12**
**SEC24A**	5q31.1	0.325	4.23E-14	**2.26E-12**
**SLC9A6**	Xq26.3	0.325	4.24E-14	**2.26E-12**
**HIPK3**	11p13	0.325	4.29E-14	**2.28E-12**
**MFAP3**	5q33.2	0.324	4.34E-14	**2.29E-12**
**ADNP2**	18q23	0.324	4.34E-14	**2.29E-12**
**NOTCH2**	1p12	0.324	4.82E-14	**2.52E-12**
**NUP58**	13q12.13	0.324	4.96E-14	**2.59E-12**
**AFDN**	6q27	0.323	5.35E-14	**2.76E-12**
**AMMECR1**	Xq23	0.323	5.64E-14	**2.89E-12**
**ZNF860**	3p23-p22.3	0.322	6.28E-14	**3.21E-12**

Table S2. The top 200 genes negatively correlated with *SPIN4*.

Correlated Gene	Cytoband	Spearman's Correlation	*p*-Value	q-Value
**PRR5**	22q13.31	-0.459	2.93E-28	**1.96E-24**
**VPS37D**	7q11.23	-0.45	4.77E-27	**2.39E-23**
**RAB6B**	3q22.1	-0.439	1.10E-25	**3.67E-22**
**RHBDD3**	22q12.2	-0.438	1.67E-25	**4.18E-22**
**DGCR6**	22q11.21|22q11	-0.434	4.22E-25	**9.40E-22**
**PARL**	3q27.1	-0.415	7.69E-23	**8.11E-20**
**MAPK12**	22q13.33	-0.412	1.69E-22	**1.54E-19**
**SLC29A4**	7p22.1	-0.41	2.81E-22	**2.35E-19**
**ST3GAL3**	1p34.1	-0.408	4.51E-22	**3.35E-19**
**SHC2**	19p13.3	-0.407	5.28E-22	**3.78E-19**
**RAB3IL1**	11q12.2-q12.3	-0.406	6.52E-22	**4.50E-19**
**NCBP2AS2**	3q29	-0.405	9.06E-22	**5.86E-19**
**COPZ2**	17q21.32	-0.404	1.16E-21	**7.26E-19**
**ASPSCR1**	17q25.3	-0.401	2.32E-21	**1.33E-18**
**SMARCD3**	7q36.1	-0.398	5.08E-21	**2.69E-18**
**EGFL7**	9q34.3	-0.397	6.94E-21	**3.48E-18**
**DPP7**	9q34.3	-0.396	9.80E-21	**4.57E-18**
**TMEM9**	1q32.1	-0.393	2.01E-20	**8.59E-18**

**GLI4**	8q24.3	-0.391	3.07E-20	**1.26E-17**
**DIPK1B**	9q34.3	-0.391	3.22E-20	**1.29E-17**
**FLAD1**	1q21.3	-0.389	5.01E-20	**1.86E-17**
**PYCR2**	1q42.12	-0.388	5.94E-20	**2.16E-17**
**TP53I13**	17q11.2	-0.388	6.57E-20	**2.35E-17**
**FTH1**	11q12.3	-0.387	8.42E-20	**2.96E-17**
**TNK2**	3q29	-0.386	9.95E-20	**3.38E-17**
**IFT27**	22q12.3	-0.386	1.03E-19	**3.45E-17**
**MRPL55**	1q42.13	-0.385	1.16E-19	**3.83E-17**
**ZNF771**	16p11.2	-0.385	1.21E-19	**3.93E-17**
**TMEM121**	14q32.33	-0.383	2.09E-19	**6.36E-17**
**ALG3**	3q27.1	-0.381	2.75E-19	**8.00E-17**
**NUDT14**	14q32.33	-0.381	2.83E-19	**8.11E-17**
**ARHGEF25**	12q13.3	-0.381	2.92E-19	**8.18E-17**
**INAFM1**	19q13.32	-0.38	3.85E-19	**1.03E-16**
**NPDC1**	9q34.3	-0.378	6.29E-19	**1.58E-16**
**SLC22A17**	14q11.2	-0.377	7.77E-19	**1.88E-16**
**MAGED4**	Xp11.22	-0.377	8.48E-19	**2.00E-16**
**SSR2**	1q22	-0.376	9.15E-19	**2.13E-16**
**ZNF579**	19q13.42	-0.376	9.63E-19	**2.22E-16**
**ETFB**	19q13.41	-0.376	9.95E-19	**2.27E-16**
**FAAP20**	1p36.33	-0.376	1.08E-18	**2.43E-16**
**DGCR6L**	22q11.21	-0.373	1.79E-18	**3.95E-16**
**SAT2**	17p13.1	-0.372	2.21E-18	**4.76E-16**
**MZT2B**	2q21.1	-0.371	2.73E-18	**5.76E-16**
**NRSN2**	20p13	-0.371	3.12E-18	**6.51E-16**
**SMIM29**	6p21.31	-0.371	3.19E-18	**6.54E-16**
**NPAS1**	19q13.32	-0.371	3.20E-18	**6.54E-16**
**SWI5**	9q34.11	-0.37	3.43E-18	**6.94E-16**
**GSC**	14q32.13	-0.37	3.78E-18	**7.57E-16**
**TRAPPC6A**	19q13.32	-0.37	4.15E-18	**8.24E-16**
**LIMS2**	2q14.3	-0.369	4.42E-18	**8.52E-16**
**LRFN1**	19q13.2	-0.369	5.10E-18	**9.73E-16**
**DUSP15**	20q11.21	-0.366	8.71E-18	**1.58E-15**
**GPC1**	2q37.3	-0.364	1.39E-17	**2.42E-15**
**CYHR1**	8q24.3	-0.363	1.63E-17	**2.75E-15**
**PRR7**	5q35.3	-0.363	1.87E-17	**3.07E-15**
**TTLL1**	22q13.2	-0.363	1.89E-17	**3.08E-15**
**CHCHD10**	22q11.23	-0.362	2.04E-17	**3.27E-15**
**NDUFB9**	8q24.13	-0.362	2.07E-17	**3.27E-15**
**LRP3**	19q13.11	-0.362	2.09E-17	**3.27E-15**
**GPR162**	12p13.31	-0.362	2.24E-17	**3.49E-15**
**COA6**	1q42.2	-0.362	2.39E-17	**3.68E-15**
**YDJC**	22q11.21	-0.361	2.58E-17	**3.94E-15**
**ABCF3**	3q27.1	-0.361	2.60E-17	**3.94E-15**
**SLC25A1**	22q11.21	-0.361	2.61E-17	**3.94E-15**
**ZDHHC1**	16q22.1	-0.361	2.87E-17	**4.30E-15**
**AP2M1**	3q27.1	-0.361	2.95E-17	**4.34E-15**
**RNF215**	22q12.2	-0.36	3.00E-17	**4.39E-15**
**MIF**	22q11.23	-0.36	3.05E-17	**4.43E-15**
**JAKMIP3**	10q26.3	-0.36	3.52E-17	**4.94E-15**

**NENF**	1q32.3	-0.359	3.89E-17	**5.38E-15**
**USE1**	19p13.11	-0.359	4.36E-17	**5.95E-15**
**FTH1P3**	2p23.3	-0.359	4.55E-17	**6.13E-15**
**KIAA0930**	22q13.31	-0.358	5.39E-17	**7.01E-15**
**MLXIPL**	7q11.23	-0.358	5.59E-17	**7.23E-15**
**PANX2**	22q13.33	-0.357	6.49E-17	**8.34E-15**
**PRRX2**	9q34.11	-0.357	6.59E-17	**8.38E-15**
**MRPL24**	1q23.1	-0.356	8.29E-17	**1.01E-14**
**RFX8**	2q11.2	-0.355	9.31E-17	**1.11E-14**
**KRTCAP2**	1q22	-0.355	9.34E-17	**1.11E-14**
**SMYD3**	1q44	-0.355	9.73E-17	**1.15E-14**
**OSGIN1**	16q23.3	-0.355	9.77E-17	**1.15E-14**
**TMEM161A**	19p13.11	-0.355	9.90E-17	**1.15E-14**
**ADSL**	22q13.1	-0.354	1.20E-16	**1.38E-14**
**FASTK**	7q36.1	-0.354	1.25E-16	**1.44E-14**
**ADGRB1**	8q24.3	-0.354	1.28E-16	**1.46E-14**
**BRSK1**	19q13.42	-0.354	1.33E-16	**1.50E-14**
**B4GALNT4**	11p15.5	-0.353	1.37E-16	**1.54E-14**
**GSTO1**	10q25.1	-0.353	1.43E-16	**1.59E-14**
**NGRN**	15q26.1	-0.353	1.62E-16	**1.76E-14**
**MRPL47**	3q26.33	-0.352	1.69E-16	**1.83E-14**
**SOX12**	20p13	-0.351	2.27E-16	**2.40E-14**
**CBX8**	17q25.3	-0.351	2.32E-16	**2.44E-14**
**PSD**	10q24.32	-0.351	2.45E-16	**2.55E-14**
**NAT14**	19q13.42	-0.35	2.73E-16	**2.81E-14**
**CDIPT**	16p11.2	-0.35	2.77E-16	**2.83E-14**
**LRRC23**	12p13.31	-0.35	2.78E-16	**2.83E-14**
**ARL8A**	1q32.1	-0.35	2.81E-16	**2.84E-14**
**LAMTOR4**	7q22.1	-0.35	2.93E-16	**2.95E-14**
**WBP1**	2p13.1	-0.349	3.27E-16	**3.25E-14**
**ADAMTS13**	9q34.2	-0.349	3.37E-16	**3.33E-14**
**PLD3**	19q13.2	-0.349	3.45E-16	**3.39E-14**
**PARVB**	22q13.31	-0.349	3.47E-16	**3.40E-14**
**ECE2**	3q27.1	-0.349	3.74E-16	**3.62E-14**
**PVT1**	8q24.21	-0.348	4.07E-16	**3.84E-14**
**C15ORF61**	15q23	-0.348	4.08E-16	**3.84E-14**
**C1ORF56**	1q21.3	-0.348	4.14E-16	**3.88E-14**
**NME4**	16p13.3	-0.347	4.61E-16	**4.30E-14**
**MAF1**	8q24.3	-0.347	4.83E-16	**4.46E-14**
**FAM118A**	22q13.31	-0.347	5.57E-16	**5.10E-14**
**ATRAID**	2p23.3	-0.346	5.68E-16	**5.17E-14**
**DTX3**	12q13.3	-0.346	5.86E-16	**5.31E-14**
**HTRA3**	4p16.1	-0.346	6.34E-16	**5.70E-14**
**C1QTNF12**	1p36.33	-0.345	7.31E-16	**6.51E-14**
**SYNDIG1**	20p11.21	-0.345	7.79E-16	**6.88E-14**
**SELENOM**	22q12.2	-0.345	8.39E-16	**7.31E-14**
**DDIT3**	12q13.3	-0.344	9.91E-16	**8.49E-14**
**ZDHHC24**	11q13.2	-0.343	1.15E-15	**9.70E-14**
**NECAB2**	16q23.3	-0.343	1.22E-15	**1.01E-13**
**FBRSL1**	12q24.33	-0.343	1.24E-15	**1.02E-13**
**MSI1**	12q24.31	-0.343	1.24E-15	**1.02E-13**

**SMARCB1**	22q11.23|22q11	-0.342	1.28E-15	**1.04E-13**
**CLEC11A**	19q13.33	-0.342	1.33E-15	**1.08E-13**
**TNFRSF18**	1p36.33	-0.342	1.42E-15	**1.14E-13**
**NUMBL**	19q13.2	-0.342	1.45E-15	**1.16E-13**
**ZNF580**	19q13.42	-0.342	1.46E-15	**1.17E-13**
**POLR2F**	22q13.1	-0.342	1.49E-15	**1.19E-13**
**B4GALT7**	5q35.3	-0.341	1.57E-15	**1.25E-13**
**P3H3**	12p13.31	-0.341	1.62E-15	**1.28E-13**
**FTL**	19q13.33	-0.341	1.84E-15	**1.43E-13**
**C12ORF57**	12p13.31	-0.34	2.03E-15	**1.56E-13**
**SELENOO**	22q13.33	-0.34	2.09E-15	**1.61E-13**
**CDPF1**	22q13.31	-0.34	2.18E-15	**1.67E-13**
**BUD23**	7q11.23	-0.339	2.46E-15	**1.87E-13**
**ZNF358**	19p13.2	-0.339	2.50E-15	**1.89E-13**
**ATXN10**	22q13.31	-0.337	3.58E-15	**2.61E-13**
**TPRA1**	3q21.3	-0.337	3.60E-15	**2.62E-13**
**NAGLU**	17q21.2	-0.337	3.72E-15	**2.69E-13**
**MAP6D1**	3q27.1	-0.337	3.94E-15	**2.84E-13**
**POLR2H**	3q27.1	-0.337	3.96E-15	**2.85E-13**
**MFRP**	11q23.3	-0.337	4.01E-15	**2.86E-13**
**TOP1MT**	8q24.3	-0.337	4.13E-15	**2.92E-13**
**PACS2**	14q32.33	-0.336	4.26E-15	**3.00E-13**
**GRP**	18q21.32	-0.336	4.43E-15	**3.09E-13**
**TTC28-AS1**	22q12.1	-0.336	4.43E-15	**3.09E-13**
**ADRA2C**	4p16.3	-0.336	4.67E-15	**3.24E-13**
**INHBE**	12q13.3	-0.336	4.70E-15	**3.25E-13**
**TCEA2**	20q13.33	-0.335	5.22E-15	**3.57E-13**
**JTB**	1q21.3	-0.335	6.05E-15	**4.08E-13**
**RTN4R**	22q11.21	-0.334	6.34E-15	**4.25E-13**
**RNF112**	17p11.2	-0.334	6.40E-15	**4.28E-13**
**PEMT**	17p11.2	-0.334	6.98E-15	**4.64E-13**
**SRRM3**	7q11.23	-0.334	7.22E-15	**4.76E-13**
**DDT**	22q11.23	-0.334	7.25E-15	**4.76E-13**
**EVA1B**	1p34.3	-0.334	7.29E-15	**4.78E-13**
**GABARAPL1**	12p13.2	-0.334	7.35E-15	**4.80E-13**
**ANAPC11**	17q25.3	-0.334	7.38E-15	**4.80E-13**
**POMC**	2p23.3	-0.333	7.80E-15	**5.06E-13**
**ENDOV**	17q25.3	-0.333	7.90E-15	**5.11E-13**
**FAHD2CP**	2q11.2	-0.333	8.04E-15	**5.18E-13**
**RHBDD2**	7q11.23	-0.333	8.16E-15	**5.24E-13**
**RNF7**	3q23	-0.333	8.27E-15	**5.30E-13**
**PLCB4**	20p12.3-p12.2	-0.333	9.21E-15	**5.79E-13**
**SLC38A10**	17q25.3	-0.332	9.93E-15	**6.22E-13**
**NAXE**	1q22	-0.331	1.15E-14	**7.19E-13**
**CD27-AS1**	12p13.31	-0.331	1.22E-14	**7.53E-13**
**PCGF1**	2p13.1	-0.331	1.32E-14	**8.07E-13**
**TSPAN7**	Xp11.4	-0.331	1.35E-14	**8.24E-13**
**PALM**	19p13.3	-0.33	1.41E-14	**8.54E-13**
**TMEM158**	3p21.31	-0.33	1.41E-14	**8.54E-13**
**CCL26**	7q11.23	-0.33	1.58E-14	**9.48E-13**
**THBS3**	1q22	-0.329	1.84E-14	**1.10E-12**

**PKN1**	19p13.12	-0.329	1.86E-14	**1.10E-12**
**RNF187**	1q42.13	-0.329	1.91E-14	**1.13E-12**
**GADD45G**	9q22.2	-0.329	1.97E-14	**1.15E-12**
**MDP1**	14q12	-0.327	2.72E-14	**1.56E-12**
**THOC5**	22q12.2	-0.327	2.80E-14	**1.59E-12**
**PRKCSH**	19p13.2	-0.326	3.01E-14	**1.69E-12**
**CRELD2**	22q13.33	-0.326	3.12E-14	**1.74E-12**
**THAP7**	22q11.21	-0.326	3.20E-14	**1.78E-12**
**INSYN1**	15q24.1	-0.326	3.33E-14	**1.84E-12**
**SEM1**	7q21.3	-0.326	3.40E-14	**1.87E-12**
**RAB34**	17q11.2	-0.326	3.48E-14	**1.90E-12**
**CCDC102A**	16q21	-0.325	3.85E-14	**2.08E-12**
**ELFN1**	7p22.3	-0.325	4.08E-14	**2.19E-12**
**CNPY3**	6p21.1	-0.324	4.32E-14	**2.29E-12**
**MXRA8**	1p36.33	-0.324	4.63E-14	**2.43E-12**
**MYL9**	20q11.23	-0.324	4.69E-14	**2.46E-12**
**SLC2A11**	22q11.23	-0.324	4.70E-14	**2.46E-12**
**PDE9A**	21q22.3	-0.324	5.03E-14	**2.61E-12**
**TMEM240**	1p36.33	-0.324	5.04E-14	**2.61E-12**
**BRI3**	7q21.3	-0.323	5.28E-14	**2.73E-12**
**GRASP**	12q13.13	-0.323	5.59E-14	**2.87E-12**
**METTL26**	16p13.3	-0.322	6.47E-14	**3.27E-12**
**SNF8**	17q21.32	-0.322	6.82E-14	**3.44E-12**
**UFSP1**	7q22.1	-0.322	6.99E-14	**3.51E-12**
**TANGO2**	22q11.21	-0.322	7.00E-14	**3.51E-12**
**SMIM7**	19p13.11	-0.322	7.02E-14	**3.51E-12**
**TMEM74B**	20p13	-0.322	7.18E-14	**3.58E-12**
**MCRIP2**	16p13.3	-0.322	7.23E-14	**3.60E-12**
**MAPKAPK5-AS1**	12q24.12	-0.321	8.46E-14	**4.17E-12**
